# Postnatal assessment for renal dysfunction in women with hypertensive disorders of pregnancy: A prospective observational study

**DOI:** 10.1007/s40620-021-01134-7

**Published:** 2021-09-24

**Authors:** Emmanouil Kountouris, Katherine Clark, Polly Kay, Nadia Roberts, Kate Bramham, Nikos A. Kametas

**Affiliations:** 1 Antenatal Hypertension Clinic, King’s College Hospital, London, UK; 2 Department of Women and Children’s Health, King’s College London, London, UK; 3 King’s Kidney Care, King’s College Hospital, London, UK; 4 Fetal Medicine Research Institute, King’s College Hospital, 16-20 Windsor Walk, London, SE5 8BB UK

**Keywords:** Pre-eclampsia, Postpartum follow-up, Renal dysfunction, Proteinuria, eGFR

## Abstract

**Background:**

Hypertensive disorders of pregnancy are associated with chronic kidney disease. Early detection of renal dysfunction enables implementation of strategies to prevent progression. International guidelines recommend review at 6–8 weeks postpartum to identify persistent hypertension and abnormal renal function, but evidence for the efficacy of this review is limited.

**Methods:**

All women attending a specialist fetal-maternal medicine clinic for hypertensive disorders of pregnancy (pre-eclampsia, chronic hypertension, gestational hypertension) were invited for a 6–8 weeks postpartum review of their blood pressure and renal function in order to establish the prevalence and independent predictors of renal dysfunction. Renal dysfunction was defined as low estimated Glomerular Filtration Rate (eGFR < 60 ml/min/1.73 m^2^) or proteinuria (24-h protein excretion > 150 mg or urinary albumin-to-creatinine ratio > 3 mg/mmol). All women attending a specialist clinic for hypertensive disorders were invited for a 6–8 weeks postpartum review of their blood pressure and renal function. Demographics, pregnancy and renal outcomes were prospectively collected.

**Results:**

Between 2013 and 2019, 740 of 1050 (70.4%) women who had a pregnancy complicated by a hypertensive disorder attended their 6–8 weeks postpartum visit. Renal dysfunction was present in 32% of the total cohort and in 46% and 22% of women with and without pre-eclampsia, respectively. Multivariate logistic regression demonstrated that independent predictors were pre-eclampsia, chronic hypertension, highest measured antenatal serum creatinine, highest measured antenatal 24-h urinary protein, and blood pressure ≥ 140/90 mmHg at the postnatal visit.

**Conclusions:**

Renal dysfunction was present in one in three women with hypertensive disorders of pregnancy at 6–8 weeks postpartum. This includes women with gestational hypertension and chronic hypertension without superimposed pre-eclampsia, and thus these women should also be offered postnatal review.

**Graphic abstract:**

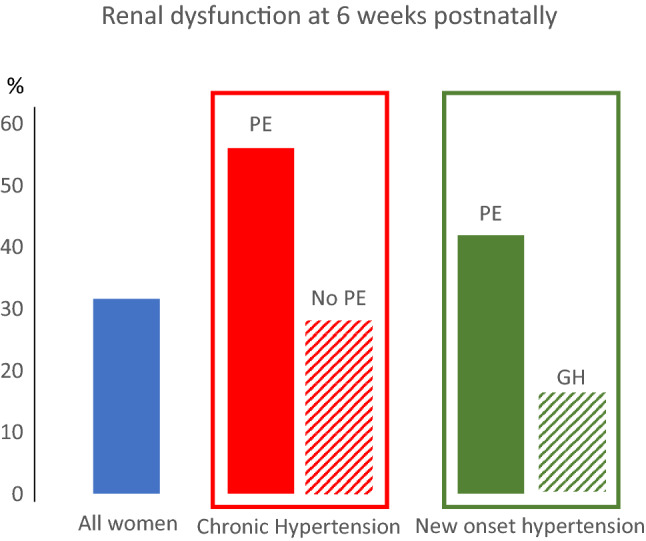

**Supplementary Information:**

The online version contains supplementary material available at 10.1007/s40620-021-01134-7.

## Introduction

Hypertensive disorders of pregnancy affect 8–12% of pregnancies worldwide and are one of the leading causes of maternal and perinatal mortality [1]. In addition to immediate risks to maternal health, hypertensive disorders of pregnancy also negatively affect future health, including cardiovascular disease with risk related to the severity of the disorder [2]. Large epidemiological studies have also reported that women with pre-eclampsia [3, 4] or any of the hypertensive disorders of pregnancy [5, 6], have up to 10-times higher risk of developing end stage kidney disease (ESKD), compared to women with uncomplicated pregnancies.

The association between the hypertensive disorders of pregnancy and future chronic kidney disease (CKD) is less clear. A meta-analysis of seven cohort studies reported that at a weighted mean of 7 years postpartum, women with previous preeclampsia have a four-fold increased risk of microalbuminuria and women with severe pre-eclampsia had an eight-fold increased risk. However, there was no difference in estimated glomerular filtration rate (eGFR) in women with previous pre-eclampsia compared to women with uncomplicated pregnancies [7]. More recently, the risk of albuminuria and CKD after pre-eclampsia was not reported to be significantly higher in a large meta-analysis but data were heterogeneous with incomplete follow-up [4]. On the contrary, data from another meta-analysis suggested that both previous pre-eclampsia and gestational hypertension were associated with increased risk of future CKD [6].

In the United Kingdom, the National Institute for Health and Care Excellence (NICE) guidelines recommend a medical review 6–8 weeks postpartum for all women with hypertensive disorders of pregnancy in order to assess future risk for cardiovascular disease and plan lifestyle modifications and appropriate follow-up, but renal assessment is advised only in women with pre-eclampsia [8]. However, due to variations in pre-eclampsia definition [9, 10], lack (of ascertainment) of confirmed diagnosis of pre-eclampsia at term and potential risk of future CKD across the spectrum of hypertensive disorders of pregnancy [6], it is unclear whether postnatal renal risk assessment is needed for all women with hypertensive disorders of pregnancy or if risk factors can be used to target those at greatest risk.

We sought to (1) establish the prevalence of reduced eGFR or proteinuria in women with different hypertensive disorders of pregnancy attending their routine postnatal visit and (2) determine which of these disorders and other risk factors are associated with low eGFR or proteinuria postpartum.

## Materials and methods

All women with hypertensive disorders of pregnancy that were managed by a fetal-maternal medicine clinic at a University maternity unit in London, UK, were invited to attend a 6–8 weeks postnatal visit in the clinic between January 2013 and March 2019. Women with known kidney disease or with proteinuria before 20 weeks’ gestation were excluded. Blood pressure was measured twice with a validated automatic device [11] and the average of the two readings was reported. Serum creatinine concentration was quantified by a hospital laboratory IDMS traceable assay [12], and eGFR was calculated using the 2009 CKD-EPI equation [13]. Postpartum assessment of urinary protein excretion was performed according to national guidelines: 24-h urine protein until 2014 [14] and subsequently by spot albumin to creatinine ratio (ACR) [15]. Urinary protein was measured by the pyrogallol red molybdate dye-binding assay, albumin with the immunonephelometric method [16] and urinary creatinine with modified Jaffe’s reaction [12].

Maternal demographic characteristics at the booking visit, as well as antenatal and postnatal laboratory and clinical parameters and pregnancy outcomes were extracted from the hospital databases. We used the maternal weight at the booking visit and not the pre-pregnancy weight because the former is objectively measured by clinicians whilst the latter may suffer reporting bias by a significant number of patients. Antenatal urinary protein excretion was assessed with 24-h urine protein. The highest measured antenatal values for 24-h urine protein, serum creatinine, aspartate aminotransferase, systolic and diastolic blood pressure were reported. Women with persistent hypertension or renal dysfunction were referred to their general practitioner or a nephrology physician (KB) for ongoing follow-up.

### Definitions

Hypertensive disorders of pregnancy were defined according to the 2018 International Society for Hypertension in Pregnancy (ISSHP-2018) recommendations [17]. Birthweight percentiles for gestational age were defined according to the Fetal Medicine Foundation charts [18].

For the purposes of analysis, 24-h urine protein excretion > 150 mg and > 500 mg were estimated to be > 3 mg/mmol and > 30 mg/mmol, respectively [14, 15]. Renal outcomes were categorised according KDIGO 2012 guidelines [19] with renal dysfunction defined as the presence of proteinuria or eGFR below 60 ml/min/1.73 m^2^, in the absence of confirmation of CKD status after 90 days. Maternal characteristics and pregnancy outcomes in women with and without renal dysfunction were compared.

Caesarean sections were categorised as emergency and elective [20]. An unplanned emergency caesarean section is when there is threat to the life of the woman or fetus and needs be performed within 75 min [20]. A planned emergency caesarean section is when there is a need for early delivery, without immediate compromise of mother or baby [20]. An elective caesarean section is when there are no maternal or fetal concerns and is planned at the end of pregnancy.

### Statistical analysis

Kolmogorov–Smirnov test was used to test for normality and data presented according to distribution. Comparison between the hypertensive disorders of pregnancy was performed with the Kruskall-Wallis or Mann–Whitney-U test for numerical data and chi-square test for categorical data. The Bonferroni correction was used to correct for multiple comparisons.

Univariate binary logistic regression was used to assess associations between antenatal and postnatal variables and renal outcomes at the 6–8 weeks postnatal visit. Multivariate logistic regression was performed to assess the independent contribution of each of these variables in the prediction of renal outcomes at the 6–8 weeks postnatal visit. Maternal parameters assessed in the logistic regression models included demographic characteristics (maternal race, chronic hypertension, use of antihypertensive medications at booking), antenatal factors (the highest recorded values for total protein excretion in 24-h urine collection, serum creatinine and aspartate transaminase, systolic and diastolic blood pressure) and postnatal factors (blood pressure ≥ 140/90 mmHg at the postnatal visit). In addition, variables related to pregnancy outcome assessed in the logistic regression models were gestational age at delivery, mode of delivery, the development of pre-eclampsia and birthweight percentile.

The area under the receiver operating characteristic curve (ROC curve) was used to graphically compare the performance of the multivariate model vs pre-eclampsia.

The study was registered as a service evaluation at King’s College Hospital NHS Foundation Trust (KCH -approval M005).

## Results

### Study population

Between 2013 and 2019, 740 of 1050 (70.4%) women with hypertensive disorders of pregnancy that were managed by a fetal-maternal medicine clinic at King’s College Hospital, London, UK attended their 6–8 weeks postpartum visit at a median (IQR) of 6.5 (5.9–7.2) weeks after delivery; 53 women were excluded because they had evidence of kidney disease or proteinuria before 20 weeks’ gestation. Of the remaining 687, 240 (35%) had received care in the specialist clinic due to chronic hypertension and 447 (65%) developed new onset hypertension during their pregnancies (Table 1, Supplementary Table 1). Pre-eclampsia complicated 284 (41%) pregnancies from the total cohort including 66 (27%) women with chronic hypertension and 218 (49%) women with new onset hypertension.

**Table 1:**
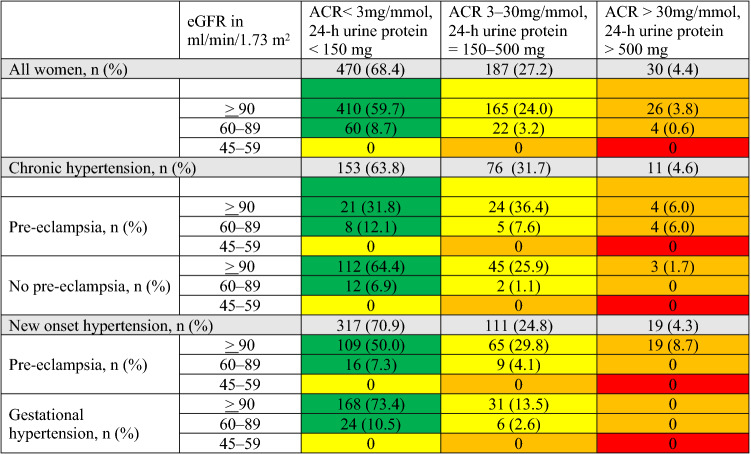
Renal function at the 6–8 weeks postnatal visit in women with hypertension in pregnancy, according to the KDIGO 2012 clinical practice guideline

The colours represent a risk stratification that determines prognosis and frequency of follow up in non-pregnant patients with chronic kidney disease. Data presented for the whole cohort, and separately for women with chronic and new-onset hypertension. For the whole cohort percentages were calculated based on the total number of women, whilst for women with chronic and new-onset hypertension, based on the number of women in each subgroup

### Features of renal dysfunction

Overall, 244 (40%) women had ACR > 3 mg/mmol (24-h protein > 150 mg) and/or eGFR ≤ 90 ml/min/1.73 m^2^ at postnatal visit (Table 1) including 217 (32%) with features of renal dysfunction: 187 (27%) A2 and 30 (4%) A3. 86 (13%) women had eGFR between 60 and 89 ml/min/1.73 m^2^ (G2) but only 26 (30%) had concurrent proteinuria (G2A2 or G2A3) (Table 1).

### Risk factors for renal dysfunction

Women with pre-eclampsia (including pre-eclampsia superimposed on chronic hypertension) were more likely to have features of renal dysfunction at the postpartum visit than women with gestational hypertension or uncomplicated chronic hypertension. However, 37 (16%) and 50 (29%) women with gestational hypertension or uncomplicated chronic hypertension, respectively, also had features of renal dysfunction at the postpartum visit (Table 1).

Comparison of maternal characteristics, pregnancy outcomes and antenatal and postnatal visit parameters between women with and without features of renal dysfunction at the postnatal visit are presented in Table 2. Women with features of renal dysfunction were more likely to be of black ethnicity and to have been prescribed antihypertensive medications at booking (Table 2) than women without features of renal dysfunction. Women with features of renal dysfunction also had earlier deliveries and smaller babies, were less likely to have had a vaginal delivery or an elective caesarean section and more likely to have had a planned emergency caesarean section.

**Table 2:** Demographic characteristics, pregnancy outcomes, antenatal and postnatal parameters of women with hypertensive disorders of pregnancy who did or did not demonstrate renal dysfunction 6–8 weeks postpartum

Variable	Without renal dysfunction* (N = 470)	With renal dysfunction* (N = 217)	p-value
Demographics			
Age, years	34.0 (31.0–37.0)	34.0 (31.0–38.)	0.962
Body mass index at 12 weeks, Kg/m^2^	27.8 (24.0–32.4)	28.4 (24.4–32.6)	0.295
Racial origin			**0.014**
White	209 (44.5%)	74 (34.1%)	**0.010**
Black	200 (42.6%)	118 (54.4%)	**0.004**
Others	61 (13.0%)	25 (11.5%)	0.591
Previous history of pre-eclampsia	137 (57.8%)	75 (66.4%)	0.125
Smoking	1 (0.2%)	2 (0.9%)	0.190
Family history of pre-eclampsia	63 (29.1%)	22 (10.1%)	0.202
Parity			0.281
Nulliparous	233 (49.6%)	104 (47.9%)	
Multiparous, no previous pre-eclampsia	100 (21.3%)	38 (17.5%)	
Multiparous, previous pre-eclampsia	137 (57.8%)	75 (34.6%)	
Past medical history			
Chronic hypertension	153 (32.6%)	87 (40.1%)	0.054
Asthma	22 (4.7%)	13 (6.0%)	0.468
Diabetes	11 (2.3%)	8 (3.7%)	0.317
Thyroid disease	15 (3.2%)	5 (2.3%)	0.520
Neurological disease	11 (2.3%)	6 (2.8%)	0.793
Antihypertensive medications at booking	58 (12.3%)	41 (18.9%)	**0.023**
Pregnancy outcomes			
Gestational age at delivery in weeks	39.0 (37.7–39.9)	38.1 (36.2–39.3)	** < 0.001**
Birthweight in grams	3112.0 (2700.0–3516.3)	2845.0 (2297.5–3323.0)	** < 0.001**
Birthweight percentile	32.36 (7.91–64.48)	21.67 (2.83–51.48)	**0.006**
Delivery Mode			** < 0.001**
Vaginal	247 (52.6%)	93 (42.%)	**0.018**
Elective caesarean section	51 (10.9%)	13 (6.0%)	**0.042**
Planned emergency caesarean section	76 (16.2%)	69 (31.8%)	** < 0.001**
Unplanned emergency caesarean section	96 (20.4%)	42 (19.4%)	0.745
Chronic hypertension—no pre-eclampsia	124 (26.4%)	50 (23.0%)	0.349
Chronic hypertension—pre-eclampsia	29 (6.2%)	37 (17.1%)	** < 0.001**
New onset hypertension—gestational hypertension	192 (40.9%)	37 (17.1%)	** < 0.001**
New onset hypertension—pre-eclampsia	125 (26.6%)	93 (42.9%)	** < 0.001**
Highest antenatal values			
24-h urine protein in mg (N = 652)	147.0 (101.0–274.0)	360.5 (164.8–989.3)	** < 0.001**
Serum creatinine in μmol/L	56.0 (50.0–64.0)	62.0 (54.0–74.0)	** < 0.001**
Aspartate aminotransferase in IU/L	26.0 (21.0–32.0)	28.0 (22.0–41.0)	** < 0.001**
Systolic blood pressure in mm Hg	147.0 (140.0–156.0)	151.0 (142.0–162.0)	**0.003**
Diastolic blood pressure in mm Hg	91.0 (82.0–97.0)	93.0 (88.0–100.0)	**0.006**
6-weeks postnatal visit			
24-h urine protein in mg (N = 304)	86.0 (66.0–108.0)	232.0 (180.0–442.0)	** < 0.001**
ACR in mg/mmol (N = 383)	1.23 (0.73–1.82)	8.39 (5.41–16.94)	** < 0.001**
ACR < 3 mg/mmol, 24-h urine protein < 150 mg	470 (100%)	0	
ACR 3–30 mg/mmol, 24-h urine protein = 150–500 mg	0	187 (86.2%)	
ACR > 30 mg/mmol, 24-h urine protein > 500 mg	0	30 (13.8%)	
eGFR-EPI in ml/min/1.73m^2^	111.9 (97.7–120.2)	113.2 (97.6–125.6)	0.058
eGFR EPI < 90 ml/min/1.73m^2^	60(12.8%)	26 (12%)	0.773
Systolic blood pressure in mm Hg	125.5 (118.5–135.0)	128.0 (117.0–139.0)	0.275
Diastolic blood pressure in mm Hg	81.0 (75.0–87.0)	81.0 (75.3–89.8)	0.266
Blood pressure ≥ 140/90 mm Hg	104 (22.1%)	67 (30.9%)	**0.014**

p-values < 0.05 were considered statistically significant and are presented with bold numbers

*Renal dysfunction is defined as ACR > 3 mg/mmol or protein excretion in a 24-h urine collection > 150 mg or eGFR < 60 ml/min/1.73 m^2^. Continuous variables are expressed as median (25th–75th percentile). Categorical variables are expressed as n (%)

The highest antenatal 24-h urine protein, serum creatinine, aspartate aminotransferase and systolic and diastolic blood pressure levels were observed in women with features of renal dysfunction, and a higher proportion of these women also had blood pressure > 140/90 mmHg at their postpartum visit compared to those without renal dysfunction.

There were no differences between maternal demographics, pregnancy outcomes and antenatal kidney function between women with eGFR > 90 and 60–89 ml/min/1.73 m^2^ without ACR > 3 mg/mmol (24-h protein > 150 mg) (Supplementary Table 2).

### Logistic regression analysis

Significant associations with features of renal dysfunction at postnatal visit identified by univariate binary logistic regression are shown in Table 3. Multivariate logistic regression demonstrated that pre-eclampsia was associated with a two-fold increased risk of features of renal dysfunction at postnatal visit compared to women with other gestational hypertension and uncomplicated chronic hypertension (Table 3). Chronic hypertension during pregnancy or having blood pressure > 140/90 mmHg at postpartum visit were also associated with increased risk of features of renal dysfunction. An increase in highest antenatal serum creatinine concentration by 1 µmol/l, and in highest antenatal protein excretion in 24-h urine collection by 100 mg increased the risk of postpartum features of renal dysfunction by 2% and 4%, respectively (Table 3). The area under the operating characteristic curve for the final multivariate model was 0.73 (95% CI 0.68–0.77), which was superior to that of a model with pre-eclampsia as the only predictor, following the NICE recommendation, 0.64 (95% CI 0.59–0.68), p < 0.001.

**Table 3:** Univariate and multivariate regression for the prediction of abnormal renal function at the 6–8 weeks postnatal visit in women with hypertensive disorders of pregnancy

Variable	B	OR (95% CI)	R^2^	p-value
Univariate regressions				
Demographics				
Race			0.018	
White				ref
Black	0.511	1.666 (1.175–2.363)		**0.004**
Others	0.146	1.158 (0.677–1.978)		0.593
Chronic hypertension	0.327	1.387 (0.994–1.935)	0.007	0.054
Antihypertensive medications at booking	0.504	1.655 (1.069–2.562)	0.010	**0.024**
Body mass index	0.0006	1.006 (0.982–1.031)	0.0004	0.63
Pregnancy outcomes				
Gestational age at delivery in weeks	− 0.153	0.858 (0.807–0.912)	0.051	** < 0.001**
Birthweight percentile	− 0.146	0.864 (0.773–0.966)	0.013	**0.010**
Delivery mode			0.046	
Vaginal				ref
Caesarean section Category 4	− 0.390	0.677 (0.352–1.302)		0.242
Caesarean section Category 3	0.880	2.411 (1.610–3.611)		** < 0.001**
Caesarean section Category 1 & 2	0.150	1.162 (0.753–1.793)		0.498
Pre-eclampsia	0.089	3.066 (2.198–4.277)	0.089	** < 0.001**
Highest antenatal values				
24-h urine protein in mg	0.001	1.001 (1.000–1.001)	0.101	** < 0.001**
Serum creatinine in μmol/L	0.038	1.039 (1.026–1.052)	0.079	** < 0.001**
Aspartate aminotransferase in IU/L	0.004	1.004 (1.000–1.008)	0.007	0.078
Systolic blood pressure in mm Hg	0.017	1.018 (1.005–1.030)	0.016	**0.005**
Diastolic blood pressure in mm Hg	0.022	1.022 (1.005–1.039)	0.013	**0.013**
6–8 weeks postnatal visit				
Blood pressure ≥ 140/90 mm Hg	0.452	1.572 (1.096–2.256)	0.012	**0.014**
Multivariate regression				
			0.192	
Pre-eclampsia	0.754	2.126 (1.403–3.224)		** < 0.001**
Chronic hypertension	0.473	1.605 (1.073–2.400)		**0.021**
Highest antenatal creatinine	0.024	1.024 (1.010–1.038)		**0.001**
Highest antenatal protein excretion in 24-h urine collection	0.000	1.0004 (1.0002–1.0006)		** < 0.001**
BP ≥ 140/90 mm Hg at postnatal review	0.512	1.668 (1.103–2.522)		**0.015**

p-values < 0.05 were considered statistically significant and are presented with bold numbers

## Discussion

Approximately one in three women with hypertensive disorders of pregnancy had features of renal dysfunction at 6–8 weeks postnatal visit. Nearly half of the women with pre-eclampsia or superimposed pre-eclampsia had features of renal dysfunction, but also one in five women with gestational hypertension of chronic hypertension without superimposed pre-eclampsia had features of renal dysfunction and would not previously have been investigated according to NICE recommendations. The majority of women had features of mild renal dysfunction (G1A2 or G1A3); however, one in eight women with hypertensive disorders of pregnancy had features of G2A2 or G2A3 with eGFR 60–89 ml/min/1.73 m^2^ postpartum. Maternal black ethnicity, antihypertensive medication at booking, mode and gestational age at delivery, low birthweight centile, pre-eclampsia, chronic hypertension, highest antenatal serum creatinine and 24-h protein excretion and postnatal hypertension were associated with abnormal postpartum kidney function, but only the latter five variables were independent predictors.

Estimates of features of renal dysfunction prevalence in women with hypertensive disorders of pregnancy are few and report variable thresholds of proteinuria at different time points postpartum, and to our knowledge there are no other studies describing features of renal dysfunction according to KDIGO criteria at the 6–8 weeks postpartum visit. One study (N = 121) reported persistent proteinuria (> 300 mg per 24 h) in 21% of women with pre-eclampsia at six weeks postpartum, that decreased to 14% and 2% at three months and two years postpartum, respectively [21]. Another study of 775 primiparous women with pre-eclampsia reported that overall, 14% had ACR > 3 mg/mmol at 4–24 months postpartum, but of those reviewed at 16–20 weeks postpartum (33% of women) had ACR > 3 mg/mmol [22]. A third study examining women with hypertensive disorders of pregnancy at six weeks postpartum, reported that 14%, 10% and 4% of women with pre-eclampsia (N = 288), superimposed pre-eclampsia (N = 30) and chronic hypertension (N = 51) had protein:creatinine ratio (PCR) > 30 mg/mmol [23]. Whilst challenging to compare with the findings from our cohort due to different proteinuria thresholds and time of assessment, together they support that pre-eclampsia and severity of antenatal proteinuria are associated with persistent postpartum proteinuria [21, 22]. However, in the latter study, none of the women with gestational hypertension (N = 94) had PCR > 30 mg/mmol at six weeks postpartum [23], whereas 16% of our cohort had ACR > 3 mg/mmol which likely reflects the lower threshold reported and the higher prevalence of women with chronic hypertension and of black ethnicity in our cohort.

The high proportion of women with gestational hypertension or chronic hypertension without superimposed pre-eclampsia with features of renal dysfunction postpartum suggests that antenatal, intrapartum or peripartum kidney injury or disease may have been under-recognised. Routine antenatal proteinuria assessment is done by urine dipstick, which has a sensitivity to diagnose antenatal proteinuria of only 40–60% [24]. It is therefore plausible that some women diagnosed with gestational or chronic hypertension did not have a formal quantification to confirm proteinuria over diagnostic threshold for pre-eclampsia as a result of false negative dipstick results. Accurate assessment of urine ACR throughout pregnancy in women with gestational hypertension and chronic hypertension could identify the proportion of women with pre-existing antenatal glomerular proteinuria which is persistent postpartum. In addition, other obstetric factors may contribute to peripartum acute kidney injury (AKI), which could have led to ensuing proteinuria after the antenatal period. Risk factors for AKI including maternal sepsis and haemorrhage frequently occur peri- or post-partum. It is likely that all women with hypertensive disorders of pregnancy are vulnerable to further peripartum renal insults which may lead to longer term kidney pathology.

It is now recognised that AKI in non-pregnant populations is associated with progression to CKD even in children and young adults, and subclinical CKD has been proposed to contribute to future pregnancy complications in women of child-bearing age [25, 26]. In our data, the highest antenatal creatinine was associated with features of renal dysfunction at the postpartum visit which suggests that AKI was present in the antenatal period. It is likely that antenatal AKI is associated with severe pre-eclampsia, as evidenced by a doubling in the risk for features of renal dysfunction by the presence of pre-eclampsia in the multivariate model.

We also report that approximately one in 12 women had eGFR < 90 ml/min/1.73m^2^ at postnatal visit, and there were no differences according to type of hypertensive disorder of pregnancy. The proportion of women with reduced eGFR was similar to that reported at more than 4 months postpartum in a study of women with new-onset pre-eclampsia[22] but it was higher than that reported by others at six weeks [23]. This may be related to higher numbers of women with chronic hypertension and of black ethnicity in our cohort, which are established risk factors for CKD [27, 28]. Reduced GFR is a recognised risk factor for the development of hypertensive disorders of pregnancy [29], and it is unknown whether reduced GFR was pre-existing prior to pregnancy and masked by gestational changes in creatinine concentration during pregnancy or previously undetected as many women were referred at time of onset of the hypertensive disorder of pregnancy.

Alternatively, renal dysfunction at the postpartum visit could be a consequence of inadequate blood pressure control after pregnancy. In keeping with others [21, 22], higher antenatal systolic and diastolic blood pressures were present in women with postpartum features of renal dysfunction, which may also reflect the severity of hypertensive disorder of pregnancy, and one in four women had blood pressure > 140/90 mmHg at postnatal assessment which may reflect inadequate treatment or be secondary to CKD.

Strengths of this study include the large number of patients, inclusion of women with all hypertensive disorders of pregnancy rather than just those with pre-eclampsia and the use of clearly defined protocols for the assessment of blood pressure and proteinuria. Limitations include the use of two different methodologies to assess proteinuria due to changes in national guidelines in the study period, which may have affected the proportion of women with values above abnormal thresholds, and the lack of long-term renal outcome data.

Pregnancy affords a unique opportunity for the assessment of future maternal health and enables early identification of reduced eGFR even in those who do not meet KDIGO criteria for CKD. In general populations, cardiovascular mortality increases with a reduction in eGFR below 90 ml/min/1.73 m^2^.[30] Longitudinal studies assessing the role of postpartum assessment of ACR and reduced eGFR to predict future CKD and cardiovascular disease are needed to determine the clinical value of this assessment.

## Supplementary Information

Below is the link to the electronic supplementary material.

Supplementary file1 (PDF 182 kb)

## Data Availability

The datasets generated and/or analysed during the current study are available from the corresponding author on reasonable request.
