# Supplementary material for: Postnatal assessment for renal dysfunction in women with hypertensive disorders of pregnancy: A prospective observational study
Source: J Nephrol. 2021 Sep 24;34(5):1641–9. doi: 10.1007/s40620-021-01134-7 (PMC8494670; doi:10.1007/s40620-021-01134-7)
Supplement: Supplementary file 1 — Supplementary file1 (PDF 182 kb) [file 40620_2021_1134_MOESM1_ESM.pdf]

Supplemental Material

Table of contents

Supplementary Table 1: Comparison of demographic characteristics, pregnancy and postnatal outcomes between the different types of hypertensive disorders of pregnancy

Supplementary Table 2: Comparison of demographic characteristics, pregnancy outcomes, and antenatal and postnatal parameters at the 6-8 weeks postnatal visit between subgroups of women with normal renal function (Group 1) and those with eGFR 60-89 ml/min/1.73m2 and no proteinuria (Group 2), proteinuria with eGFR>90 ml/min/1.73m2 (Group 3) and proteinuria with eGFR 60-89 ml/min/1.73m2 (Group 4).

Supplementary Table 1: Comparison of demographic characteristics, pregnancy and postnatal outcomes between the different types of hypertensive disorders of pregnancy.

Variable	Chronic hypertension no PE (N=174)	Chronic hypertension PE (N= 66)	New onset hypertension GH (N=229)	New onset hypertension PE (N= 218)	p- value
**Demographics**					
Age, years	35.5 (32.0-39.0)	35.0 (32.7-40.2)	34.0 (31.0- -37.0)	33.0 (29.0- 37.0)	**<0.001**
Body mass index at 12 weeks, Kg/m^2	30.0 (26.1- 34.7)	31.0 (26.2- 35.0)	26.8 (23.2- 31.2)	26.3 (23.4- 31.0)	**<0.001**
Racial origin					**<0.001**
White	46 (26.4%)	20 (30.3%)	125 (54.6%)	92 (42.2%)	
Black	106 (60.9%)	42 (63.6%)	75 (32.8%)	95 (43.6%)	
Others	22 (12.6%)	4 (6.1%)	29 (12.7%)	31 (14.2%)	
Parity					
Nulliparous	63 (36.2%)	26 (39.4%)	110 (48.0%)	138 (63.3%)	**<0.001**
Multiparous, no previous PE	70 (40.2%)	24 (36.4%)	53 (23.1)	41 (18.8%)	**<0.001**
Multiparous, previous PE	65 (58.6%)	32 (80.0%)	72 (60.5%)	43 (53.8%)	**0.045**
Past medical history					
Asthma	17 (9.8%)	3 (4.5%)	6 (2.6%)	9 (4.1%)	**0.011**
Diabetes	8 (4.6%)	4 (6.1%)	3 (1.3%)	4 (6.1%)	0.062
Thyroid disease	5 (2.9%)	4 (6.1%)	7 (3.1%)	4 (1.8%)	0.358
Neurological disease	6 (3.4%)	2 (3.0%)	5 (2.2%)	4 (1.4%)	0.749
**Pregnancy outcome**					
Gestational age at delivery in weeks	39.0 (38.1- 39.7)	37.4 (33.7-38.9)	39.5 (38.7-40.1)	37.6 (36.4- 39.0)	**<0.001**
Birthweight in grams	3140.0 (2710.0- 3460.0)	2747.0 (1805.0- 3077.5)	3285.0 (2855.5- 3589.0)	2780.0 (2189.5- 3302.0)	**<0.001**
Birthweight centile	32.1 (10.4- 61.6)	13.7 (2.8-35.3)	40.8 (10.7-68.7)	20.5 (1.4- 53.6)	**<0.001**
Estimated fetal weight <10th centile	43 (24.7%)	28 (42.4%)	54 (23.6%)	92 (42.2%)	**<0.001**
Delivery Mode					**<0.001**
Vaginal	83 (47.7%)	25 (37.9%)	131 (57.2%)	101 (46.3%)	
Caesarean section Category 4	23 (13.2%)	5 (7.6%)	27 (11.8%)	9 (4.1%)	
Caesarean section Category 3	30 (17.2%)	29 (43.9%)	21 (9.2%)	65 (29.8%)	
Caesarean section Category 1 & 2	38 (21.8%)	7 (10.6%)	50 (21.8%)	43 (19.7%)	
**6-8 weeks postnatal visit**					
eGFR-EPI in ml/min/1.73m^2	112.0 (99.2- 123.1)	103.7 (89.8- 124.3)	112.3 (97.1- 121.7)	113.4 (99.1- 122.4)	0.106
ACR in mg/mmol	1.78 (0.87- 4.29)	6.04 (1.70- 15.24)	1.67 (0.81- 2.90)	3.48 (1.25- 12.87)	**<0.001**
24h urine protein in mg	102.0 (75.5- 147.5)	122.0 (78.2- 266.5)	85.5 (63.5- 111.0)	109.0 (78.7- 182.0)	**<0.001**
Systolic blood pressure in mmHg	132.7 (126.2- 141.0)	134.7 (122.6- 142.6)	124.0 (117.2- 131.0)	122.0 (114.0- 130.0)	**<0.001**
Diastolic blood pressure in mmHg	86.5 (80.5- 92.0)	84.0 (78.9- 93.6)	80.0 (75.0- 85.0)	78.0 (71.0- 84.0)	**<0.001**

Continuous variables are expressed as median (25th–75th percentile). Categorical variables were expressed as n (%). PE: pre-eclampsia, GH: gestational hypertension, eGFR: estimated glomerular filtration rate, ACR: albumin to creatinine ratio, IQR: interquartile range

Supplementary Table 2: Comparison of demographic characteristics, pregnancy outcomes, and antenatal and postnatal parameters at the 6-8 weeks postnatal visit between subgroups of women with normal renal function (Group 1) and those with eGFR 60-89 ml/min/1.73m^2 and no proteinuria (Group 2), proteinuria with eGFR>90 ml/min/1.73m^2 (Group 3) and proteinuria with eGFR 60-89 ml/min/1.73m^2 (Group 4).

Variable	Group1 (N= 410)	Group2 (N= 60)	Group3 (N= 191)	Group4 (N= 26)	P- value
**Demographics**					
Age, years	34.0 (31.0-37.0)	35.0 (32.0- 39.75)	33.0 (30.0- 38.0)	35.0 (32.75- 38.0)	0.084
Body mass index at 12 weeks, Kg/m^2	27.8 (23.8- 32.6)	26.8 (24.5- 31.2)	28.0 (24.4- 32.4)	30.1 (24.1- 33.4)	0.610
Racial origin	* †	†			**0.001**
White	172 (42%)	37 (61.7 %)	62 (32.5%)	12 (46.2 %)	
Black	178 (43.4 %)	22 (36.7%)	107 (56%)	11 (42.3)	
Others	60 (14.6%)	1 (1.7%)	22 (11.5%)	3 (11.5%)	
Parity					0.395
Nulliparous	204 (49.8%)	29 (48.3%)	89 (46.6%)	15 (57.7%)	
Multiparous, no previous pre-eclampsia	83 (20.2%)	17 (28.3 %)	35 (18.3%)	3 (11.5%)	
Multiparous, previous pre-eclampsia	123 (30%)	14 (23.3%)	67 (35.1%)	8 (30.8%)	
Past medical history					
Chronic hypertension	133 (32.4%)	20 (33.3%)	76 (93.8%)	11 (42.3%)	0.285
Asthma	20 (4.9%)	2 (3.3%)	11 (5.8%)	2 (7.7%)	0.810
Diabetes	11 (2.7%)‡	0‡	5 (2.6%)‡	3 (11.5%)	**0.027**
Thyroid disease	13 (3.2%)	2 (3.3%)	5 (2.6%)	0	0.808
Neurological disease	10 (2.4%)	1 (1.7%)	6 (3.1%)	0	0.759
Antihypertensive medications at booking	49 (12.0%)	9 (15.0%)	36 (18.8%)	5 (19.2%)	0.135
**Pregnancy outcome**					
Gestational age at delivery in weeks,	39.0 (37.7- 39.9) † ‡	38.9 (37.8- 39.8) ‡	38.3 (36.3- 39.3) ‡	37.4 (36.0- 39.1)	**<0.001**
Birthweight in grams	3117.5 (2695.0- 3507.0) †	3070.0 (2794.0- 3592.5) †	2845.0 (2310.0- 3326.0)	2757.5 (2291.3- 3288.8)	**<0.001**
Birthweight centile	31.3 (7.7- 63.8) †	33.0 (10.6- 68.5)	21.7 (2.0- 53.0)	20.3 (4.4- 49.2)	**<0.001**
Delivery Mode	† ‡				**0.001**
Vaginal	217 (52.9%)	30 (50%)	80 (41.9%)	13 (50%)	
Caesarean section Category 4	45 (11.0%)	6 (10.0%)	11 (5.8%)	2 (7.7%)	
Caesarean section Category 3	62 (15.1%)	14 (23.3 %)	60 (31.4%)	9 (34.6%)	
Caesarean section Category 1 & 2	86 (21.0%)	10 (16.7%)	40 (20.9%)	2 (7.7%)	
Pre-eclampsia	130 (31.7%) † ‡	24 (40%)† ‡	112 (58.6%)	18 (69.2%)	**0.000**
**Highest antenatal values**					
24h urine protein in mg	144.0 (101.0- 272.5) †‡	170.0 (108.0- 318.5) † ‡	367.5 (161.8- 1054.5) ‡	339.0 (168.8- 564.0)	**0.000**
Creatinine in µmol/L	55.0 (49.0-63.0) * † ‡`	63.0 (58.0-71.8)	60.0 (54.0- 72.0) ‡	74.0 (62.5- 83.0)	**0.000**
Aspartate aminotransferase in IU/L	25.0 (21.0-32.0) † ‡	27.0 (20.3- 33.8)	28.0(22.0-40.0)	32.0 (22.8-56.5)	**0.001**
Systolic blood pressure in mmHg	147.0 (140.0- 158.0)	147.0 (142.0- 152.0)	150.0 (141.0- 162.0)	156.0 (145.8- 161.3)	**0.010**
Diastolic blood pressure in mmHg	91.0 (84.5- 97.0)	91.5 (84.0- 96.0)	92.0 (87.0- 99.0)	94.0 88.8- 100.5)	**0.030**

6-weeks postnatal visit					
eGFR-EPI in ml/min/1.73m^2	113.8 (103.0- 122.5)	84.2 (77.0- 87.1)	116.0 (105.2- 126.8)	84.9 (78.0- 87.0)	
ACR in mg/mmol	1.25 (0.76-1.77)	0.98 (0.57- 2.29)	8.39 (5.27- 17.39)	8.29 (5.65- 12.70)	
24h urine protein in mg	86.0 (65.8- 108.0)	85.0 (67.0- 107.0)	232.0 (182.5- 444.5)	246.5 (169.5- 504.0)	
Systolic blood pressure in mmHg	126.1 (119.0- 135.0)	121.5 (115.1- 135.8)	128.0 (117.0- 138.5)	132.0 (113.9- 141.2)	0.254
Diastolic blood pressure in mmHg	81.0 (75.5- 87.5)	78.5 (71.5- 85.4)	81.0 (75.5- 90.0)	84.8 (72.8- 88.0)	0.273

Vs Group2: ***,** vs group3: †**,** vs Group4: ‡

Continuous variables are expressed as median (25th–75th percentile). Categorical variables were expressed as n (%).
